# Supplementary material for: Structure of the initiation-competent RNA polymerase I and its implication for transcription
Source: Nat Commun. 2016 Jul 15;7:12126. doi: 10.1038/ncomms12126 (PMC4947174; doi:10.1038/ncomms12126)
Supplement: Supplementary Information — Supplementary Figures 1-9, Supplementary Tables 1-4 and Supplementary References [file ncomms12126-s1.pdf]

Supplementary data and figures (Pilsl et al.)

Supplementary Figures

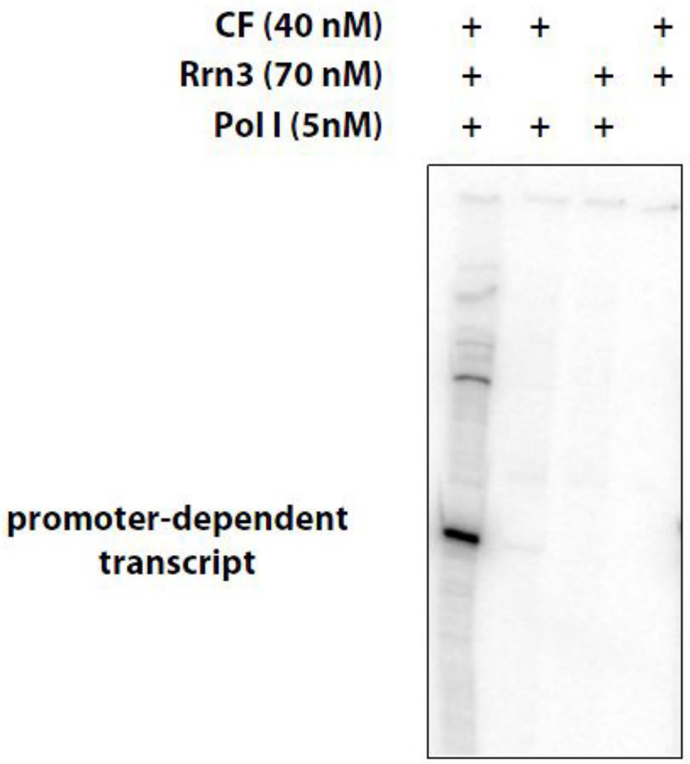

Supplementary Fig. 1: Reconstitution of promoter-dependent transcription.

Purified Pol I from strain y2423 was transcribed with or without recombinant purified CF and/or Rrn3 using the Pol I promoter-containing and linearized plasmid pMAX. Transcription was performed as described in the Methods part.

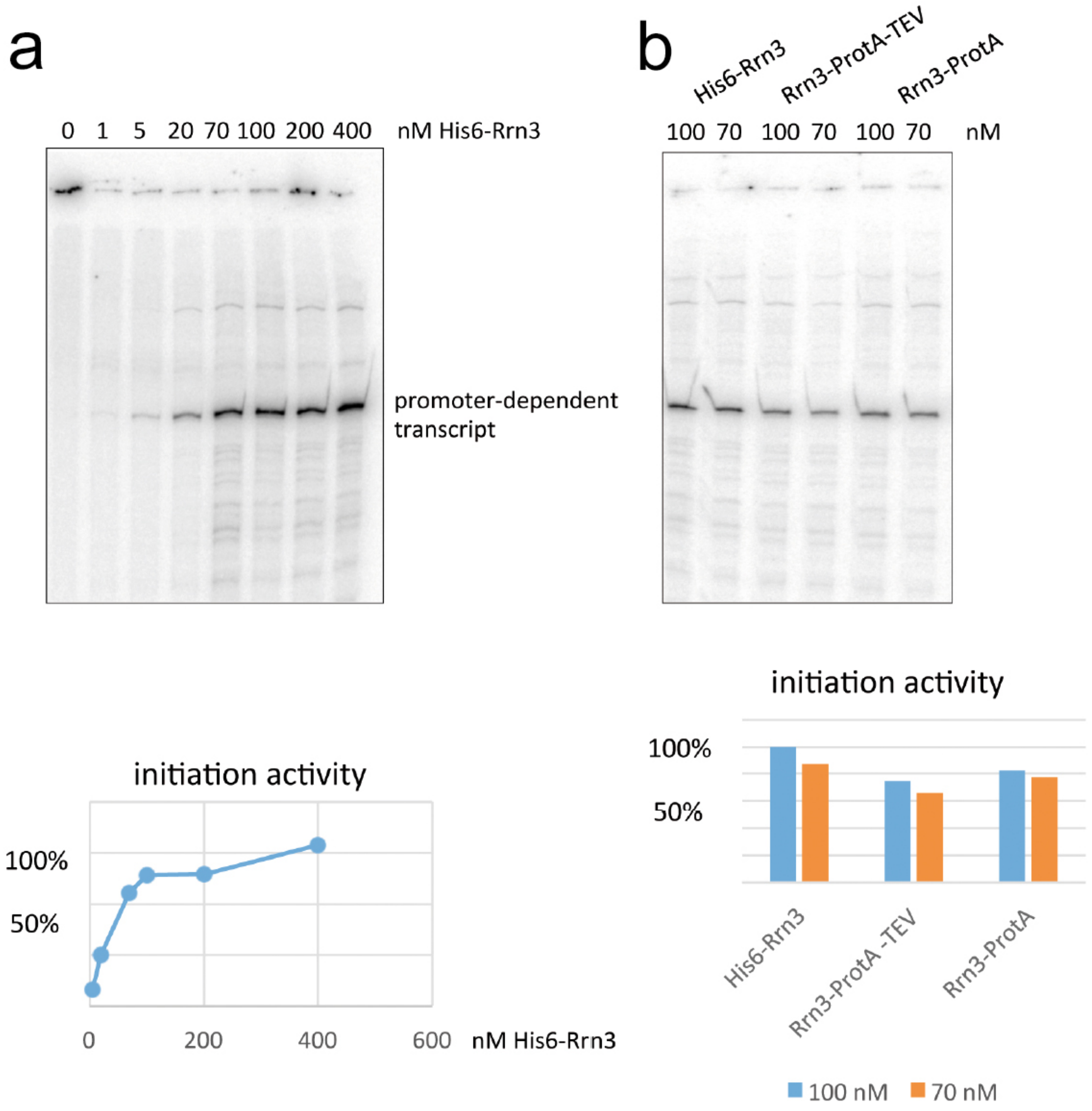

Supplementary Fig. 2: Titration of recombinant Rrn3 containing different epitope tags to constant amounts of Pol I and CF. Rrn3 proteins were expressed in E.coli, purified and their concentration was determined. a) Rising amounts of His3-Rrn3 were used in promoter-dependent transcription containing 5 nM Pol I and 40 nM CF. b) Comparison of different tagged Rrn3 proteins in promoter dependent transcription containing 5nM Pol I and 40 nM CF.

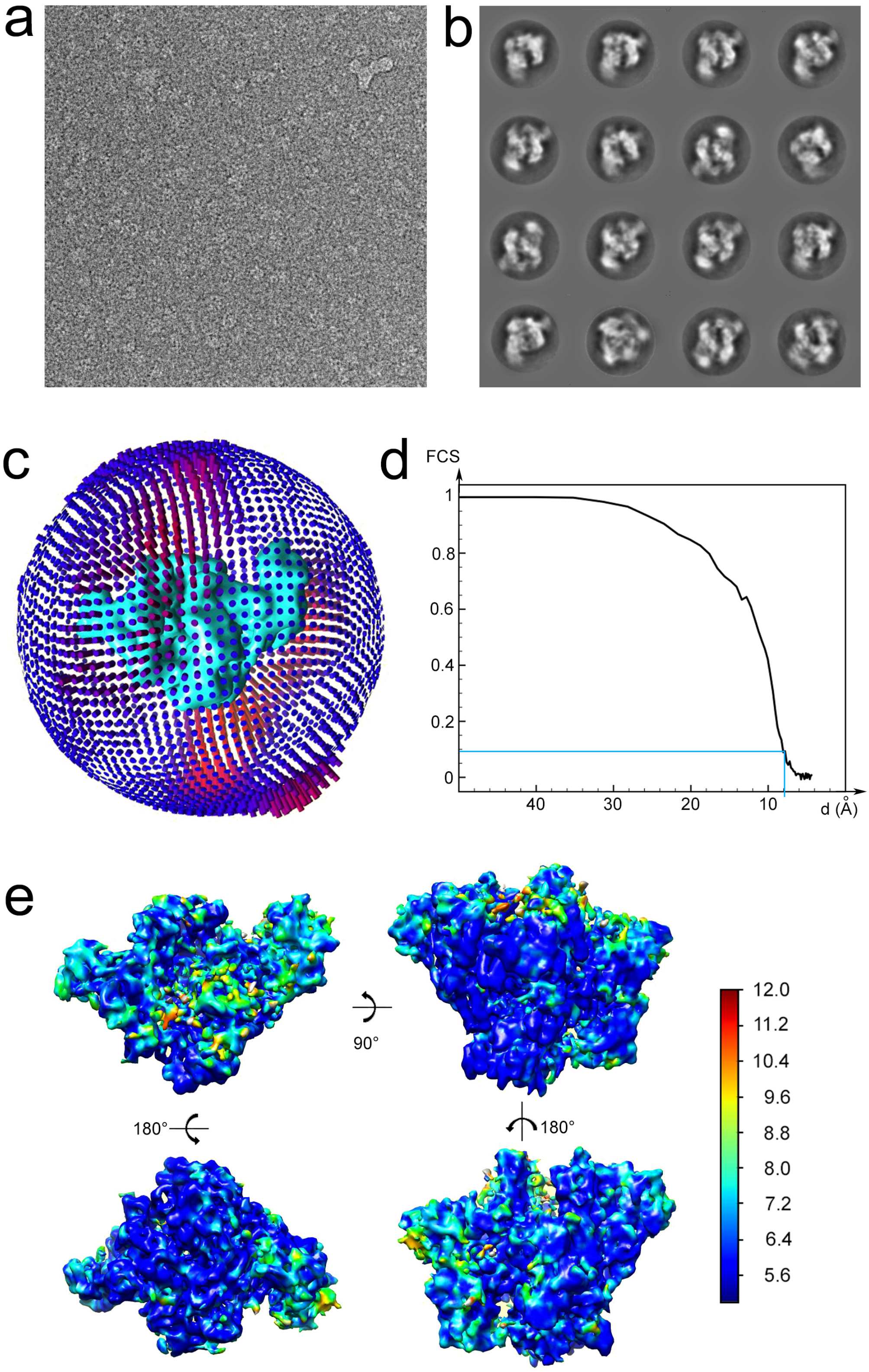

Supplementary Fig. 3: Structural analysis of the Pol I-Rrn3 complex.

a) Original images of the frozen-hydrated Pol I –Rrn3 complex adsorbed on a thin carbon film. b) Two-dimensional class averages obtained using the relion software. c) Angular distribution of the images used for 3-D reconstruction. d) Fourier Shell Correlation curve as a function of distance in Å. e) Local resolution map obtained using the Resmap software.

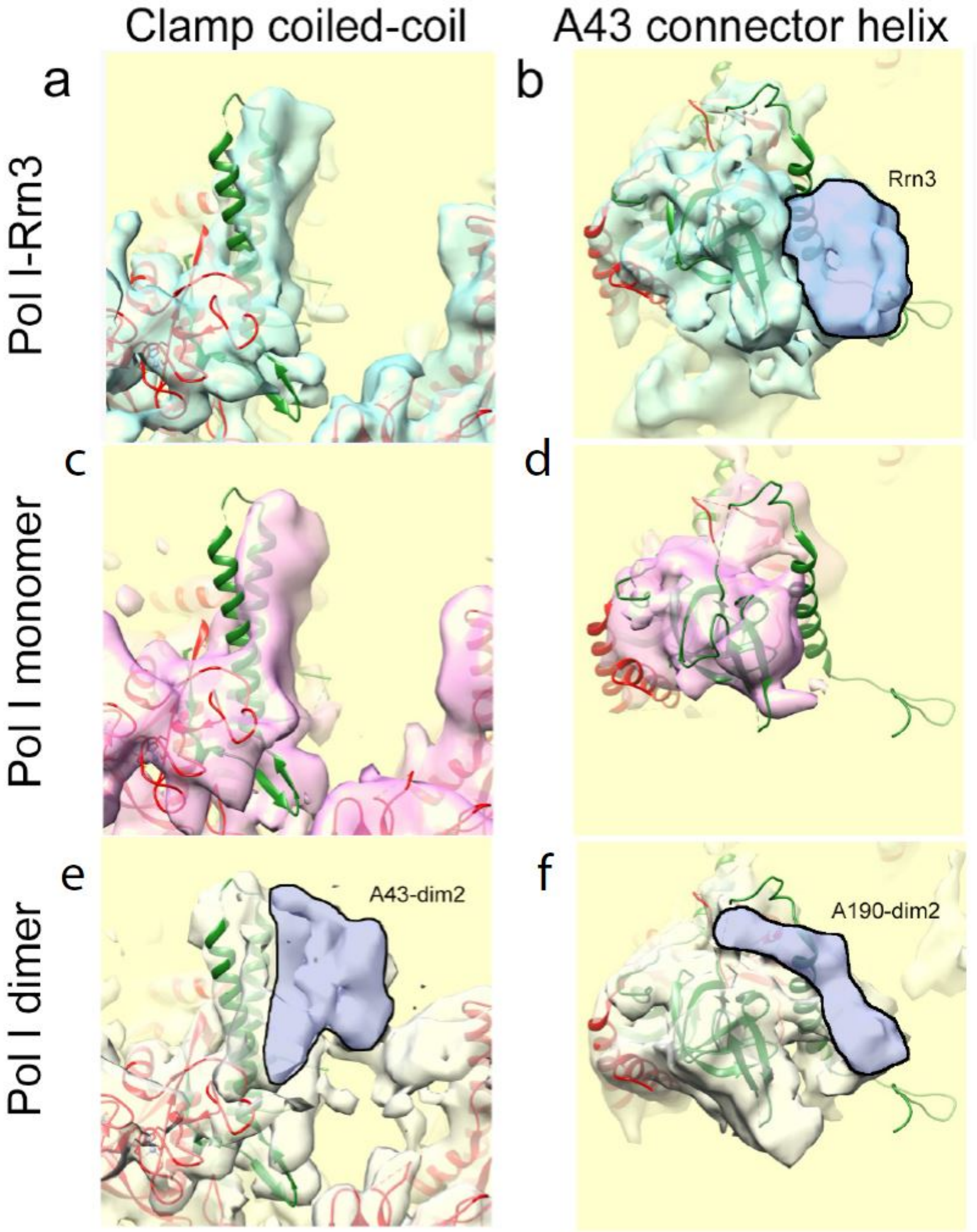

Supplementary Fig. 4: Comparison of key structural features in different Pol I conformational states.

Positions of the clamped coil domains (subunit A190) (a, c, e) and connector helix (subunit A43) (b, d, f) are shown in green in the cryo-EM maps of the Pol I- Rrn3 complex (a, b), the Pol I monomer (c, d) and the Pol I dimer (e, f). Electron dense parts of Rrn3 or the dimerizing Pol I (A43-dim2, A190-dim2) molecule are indicated in light blue.

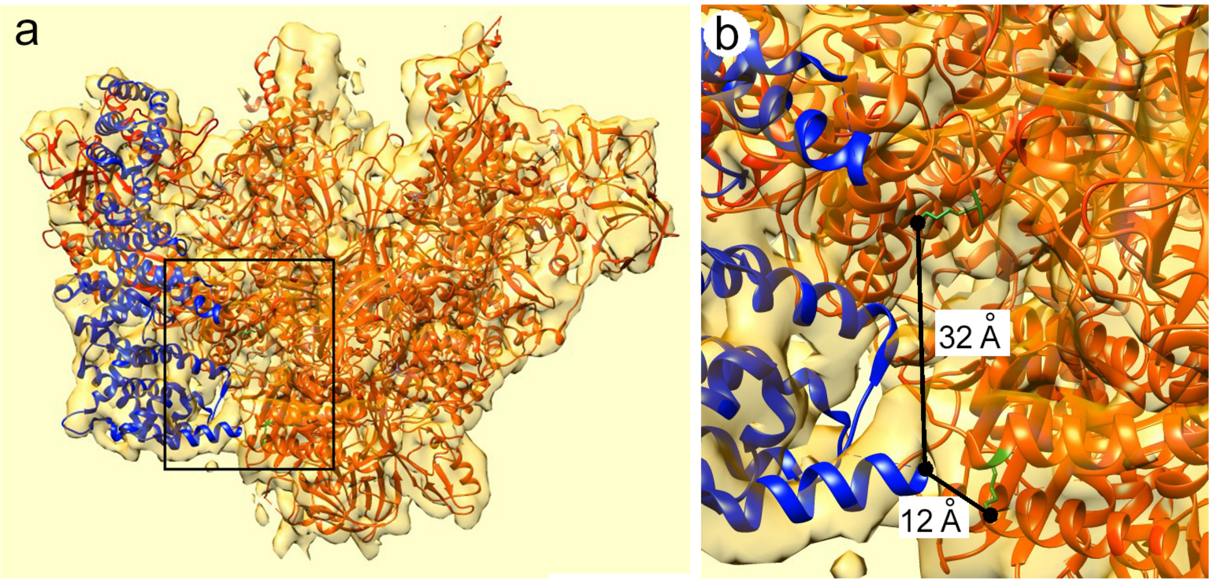

Supplementary Fig. 5: Localization of BS3 cross-links between Pol I and Rrn3 as identified in [1]

Two bis-sulfosuccinimidyl-suberate intersubunit cross-linked lysine pairs were identified in the Rrn3/pol I interface, within the area highlighted in a) and enlarged in b). The only reactive Rrn3 lysine 558 is located within an unresolved loop. The distances measured between the basis of the loop and the reactive AC40 lysine 329 (12 Å) and A190-lysine 582 (32 Å) are within reach of the crosslinker and are thus consistent with our model.

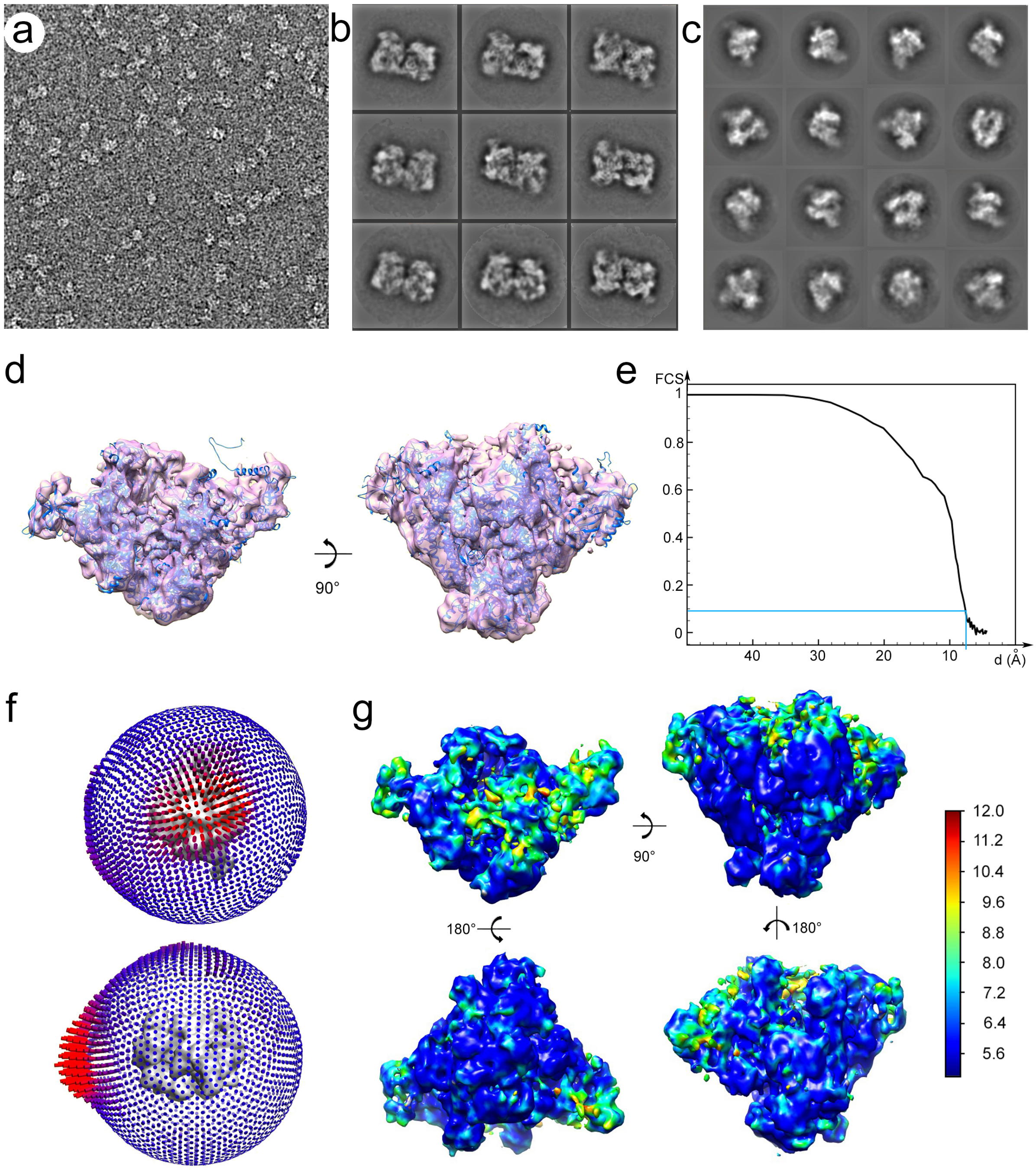

Supplementary Fig. 6: CryoEM structure of the monomeric Pol I.
a) Highly defocused original image of the frozen-hydrated Pol I molecules adsorbed on a thin carbon film. Pol I dimers and monomers can be distinguished. b) Two-dimensional class averages of Pol I dimers c) Two-dimensional class averages of Pol I monomers. d) Atomic structure of Pol I (blue ribbons) docked into the cryo-EM map of the Pol I monomer (pink envelope). e) Fourier Shell Correlation curve of the Pol I dimer dataset as a function of distance in Å. f) Angular distribution of the images used for 3-D reconstruction. g) Local resolution map obtained using the Resmap software.

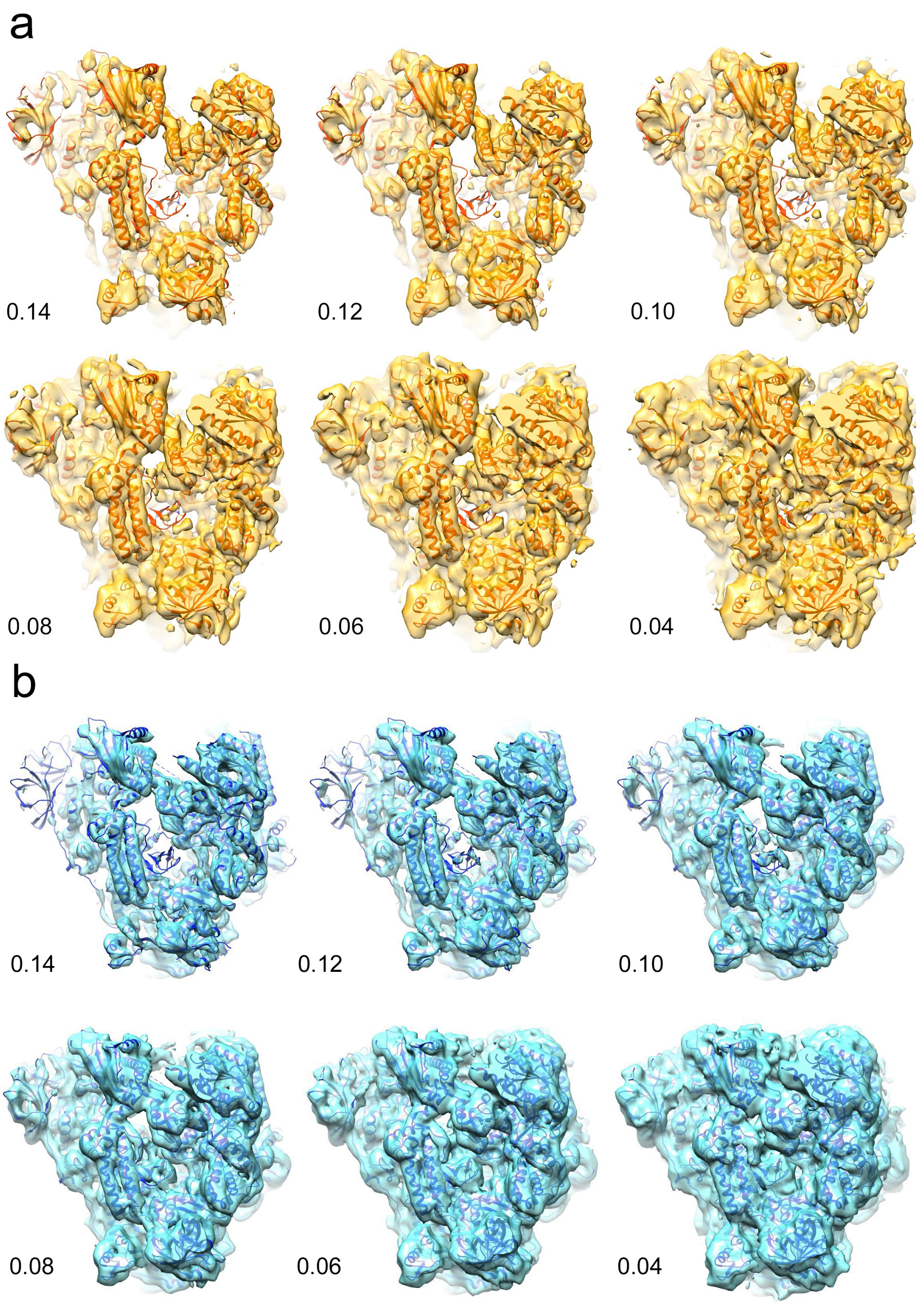

Supplementary Fig. 7: View of the Pol I atomic structure docked into the cryo EM model of the PolI-Rrn3 complex (a) and of the Pol I dimer (b) along the nucleotide entry pore and the C-terminus of A12.2 (center of the maps). The threshold of applied onto the cryo-EM maps has been lowered from 0.14 to 0.04 to visualize the progressive filling of the pore by a density similar in shape to the C-terminus of A12.2 in the case of the Pol I dimer while the pore remains free in the case of the Pol I-Rrn3 complex

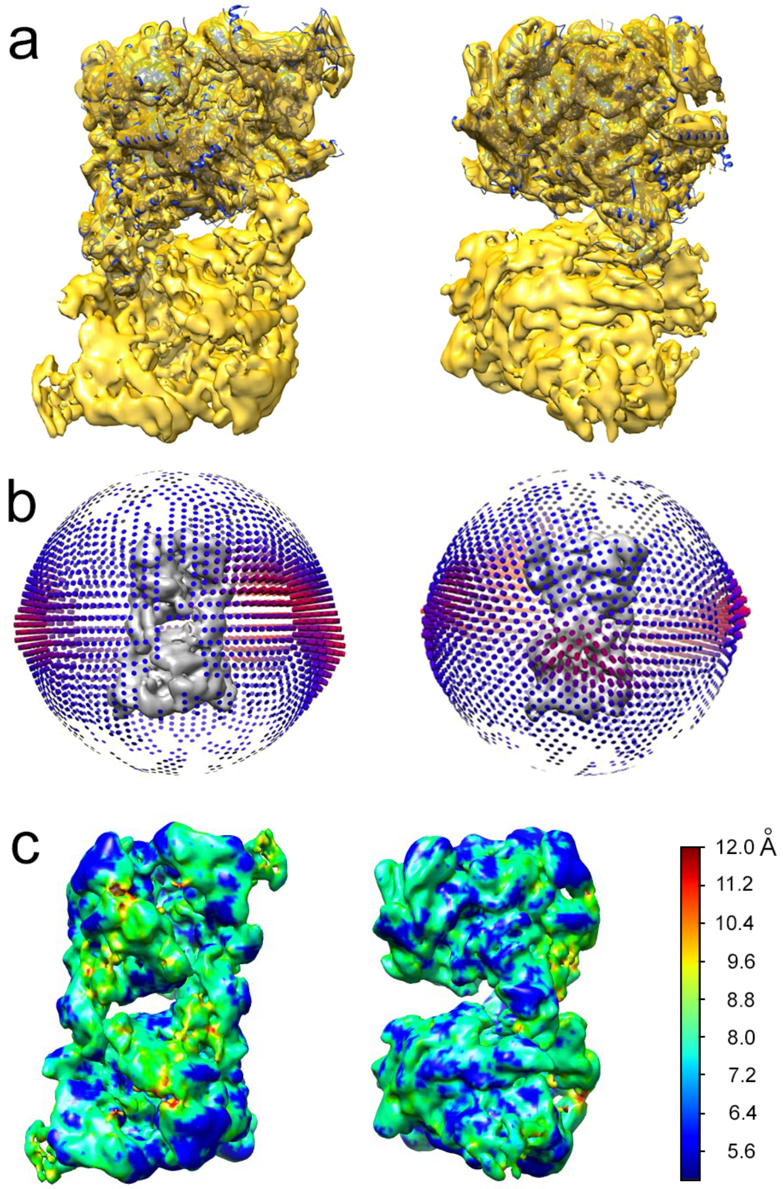

Supplementary Fig. 8: CryoEM structure of the dimeric form of Pol I.
a) Atomic structure of Pol I (blue ribbons) docked into the cryo-EM map of the Pol I dimer (yellow envelope). b) Angular distribution of the images used for 3-D reconstruction. c) Local resolution map.

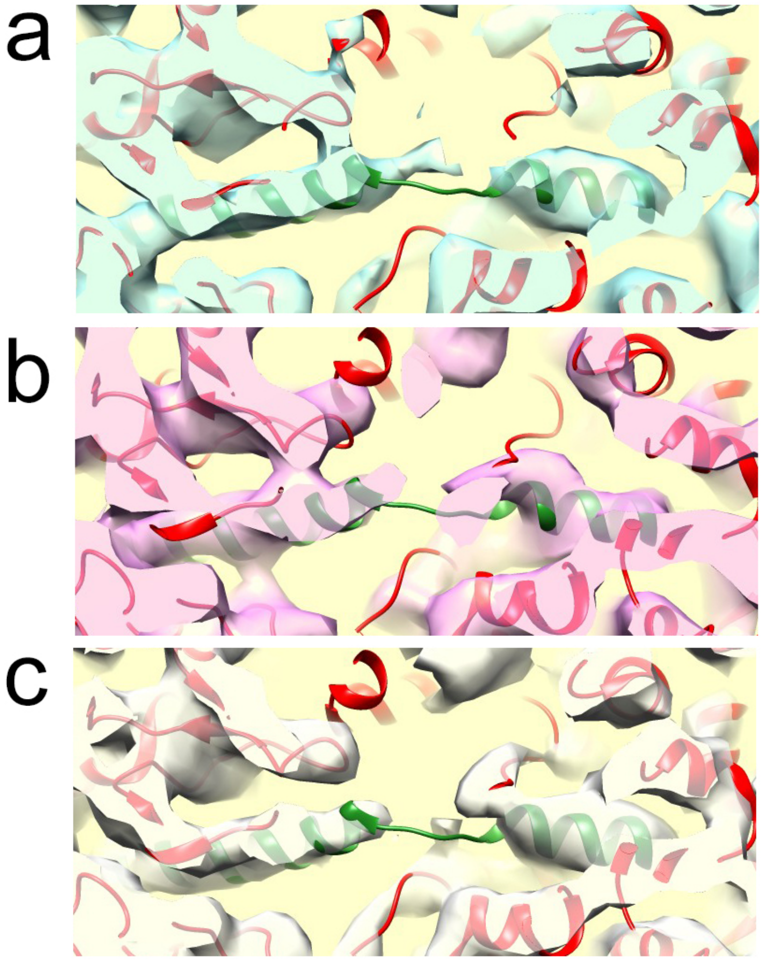

Supplementary Fig. 9: Structure of the bridge helix in different Pol I conformational states.
Shape of the locally unfolded bridge helix in the cryo-EM maps of the Pol I- Rrn3 complex (a), the Pol I monomer (b) and the Pol I dimer (c).

Supplementary Tables

Table 1: Strains

Strain	Name	Parental strain	Genotype	Reference
y2423	BY4741 A135-TEV-ProtA	BY4741	MATa, *his3-1 leu2-0 met15-0 ura3-0 rpa135::RPA135-TEV-ProtA-kanMX6*	[2]
y2183	BSY420-Rrn3-TEV-ProtA-His7-Tag	BSY420	MATa, , *ade2-1 ura3-1 trp1-1 leu2-3,112 his3-200 RRN3-TEV-ProtA-7HIS*	[3]
Y2670	yJPF162-1a	BY4741	*his31; leu20; met15-0; ura3-0; RPA135-TEV-ProtA::kanMX6 HIS3MX::GAL::HA-RPA49*	this study

Table 2: Plasmids

435	pMAX1	SacI-fragment (3780 bp) from YepSIRT [4] containing ETS1 including Pol I promoter and 520 bp at the C-terminus of 25S rDNA cloned in pBluescript (6731 bp)	this study
2145	pUC 19 tail g- TER elongated	Tailed template DNA containing 18 S rDNA for unspecific in vitro transcription	[3]
729	Ycplac111-GAL-Rrn3-TEV-ProtA-HIS	GAL-dependent Rrn3 overexpression vector (Amp; LEU)	[3]
2250	pET28b A49/34.5	expression vector for A49/34.5 heterodimer	[5]
2400	pET28b His6-Rrn3	expression vector for Rrn3	[1]
2401	pET Duet CF	expression vector for CF	[6]
2402	pET24a Rrn3-TEV-ProtA-His7	expression vector for Rrn3	this study

2403	pET28b His6-A49 186-415 (tWH)	expression vector for His6-A49 186-415 (tWH)	this study
2404	pET28b His6-A49 111-415	expression vector for His6-A49 111-415 (tWH)	this study
2405	pET28b A34.5-His6	expression vector for A34.5-His6	this study
2406	pET28b A34.5/A49 (1-186) -His6	expression vector for A34.5/A49 (1-186) -His6	this study
2407	pET28b A34.5/A49 (1-110) -His6	expression vector for A34.5/A49 (1-110) -His6	this study

Table 3: Oligonucleotides

2973	UE fwd	AGCTTAAATTGAAGTTTTTCTC	amplification of upstream element
2976	CE fwd	TAGTTTGTAATGGGAGGGG	amplification of core element
4176	#pMax rev 320 nt	ttactattgcggtaacattcat	amplification of trx template pMAX1 with#2973/2976
4177	#pMax rev 480 nt	atattgtgtggagcaaagaaat	amplification of trx template pMAX1 with#2973/2976
4178	A34.5_NcoI_fwd	ttttccatgggaATGGGCTCCAAGCTTTCG	cloning of A34.5/A49 1-186 in pET28b via NcoI/NotI PCR from plasmid#2250
4179	A34.5 NotI rev	ttttgcggccgcATCTCTATGTTTCTTTTT CTTA	cloning of A34.5 in pET28b via NcoI/NotI PCR from plasmid#2250
4180	A49_1-186 NotI_rev	ttttgcggccgctctatcgttggaagtaattt	cloning of A34.5/A49 1-186 in pET28b via NcoI/NotI PCR from plasmid#2250
4181	A49 1-110 NotI rev	ttttgcggccgctggaccccttagattctt	cloning of A34.5/A49 1-111 in pET28b via NcoI/NotI PCR from plasmid#2250
4182	A49 111 tWH Nhe1 fwd	ttttgctagcaaaataaaaagtaagagtgatactcg	cloning of A49 111-415 in pET28b via NheI/HindIII PCR from plasmid #2250

4183	A49 NdeI fwd	ttttcatatgatgtccgtgaaaaggtctgtt	Cloning of A49 in pET24a via NotI/NdeI PCR from plasmid #2250
4184	A49 Not1 rev	tttgcggccgcacgtcttggacctcttcct	cloning of A49 in pET24a via NotI/NdeI PCR from plasmid #2250
4185	A49 tWH NheI fwd	ttttgctagcccattagccaatatcgatgc	cloning of A49 tWH in pET28b via HindIII/NheI PCR from plasmid #2250
4186	A49 tWH HindIII rev	ttttaagcttctaacgtcttggacctcttc	cloning of A49 tWH in pET28b via HindIII/NheI PCR from plasmid #2250

Table 4: Ratio of Pol I monomers and dimers in dependency on Rrn3 incubation determined by EM analyses

incubation time	- fold excess Rrn3	dimers	monomers	% dimers	% monomers	% Pol I in dimers	% Pol I in monomers
30 min	0.5	168	367	31.4	68.6	47.8	52.2
	1	331	595	35.7	64.3	52.7	47.3
	5	346	602	36.5	63.5	53.5	46.5
	10	197	389	33.6	66.4	50.3	49.7
120 min	1	2586	4992	34.1	65.9	50.9	49.1
	5	2687	4876	35.5	64.5	52.4	47.6
120 min	10	286	592	32.6	67.4	49.1	50.9
	10	426	677	38.6	61.4	55.7	44.3

Incubation of recombinant Rrn3 with Pol I was performed in buffer B200 at 4°C

Supplementary References

1. Blattner, C. et al. Molecular basis of Rrn3-regulated RNA polymerase I initiation and cell growth. *Genes Dev* **25**, 2093-105 (2011).
2. Hierlmeier, T. et al. Rrp5p, Noc1p and Noc2p form a protein module which is part of early large ribosomal subunit precursors in S. cerevisiae. *Nucleic Acids Res* **41**, 1191-210 (2013).
3. Merkl, P. et al. Binding of the termination factor nsi1 to its cognate DNA site is sufficient to terminate RNA polymerase I transcription in vitro and to induce termination in vivo. *Mol Cell Biol* **34**, 3817-27 (2014).
4. Musters, W. et al. Linker scanning of the yeast RNA polymerase I promoter. *Nucl. Acids Res.* **17**, 9661-9678 (1989).
5. Geiger, S.R. et al. RNA polymerase I contains a TFIIF-related DNA-binding subcomplex. *Mol Cell* **39**, 583-94 (2010).
6. Knutson, B.A., Luo, J., Ranish, J. & Hahn, S. Architecture of the Saccharomyces cerevisiae RNA polymerase I Core Factor complex. *Nat Struct Mol Biol* **21**, 810-6 (2014).

Transcription gels used for Fig. 6

transcription gel Fig. 6a

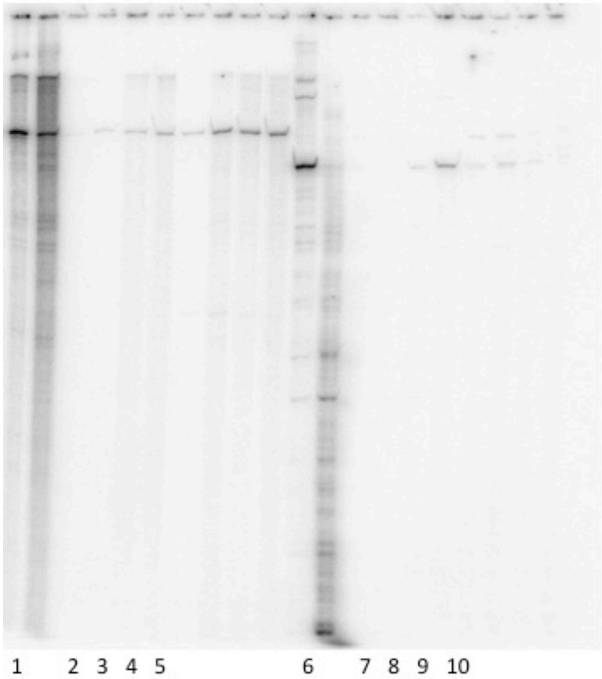

transcription gel Fig. 6b

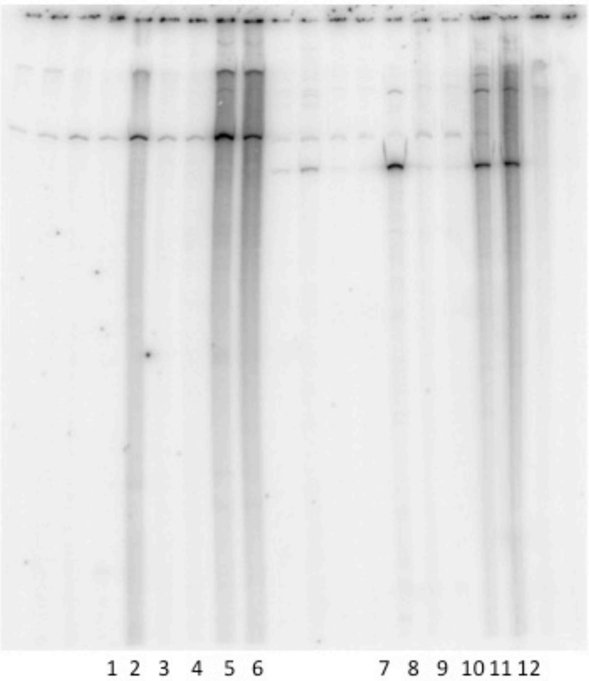

The lanes depicted in Fig. 6a and b are indicated
